# Evidence gap in predicting intracranial haemorrhage risk in people with glioma on anticoagulants: a scoping review

**DOI:** 10.1186/s12883-025-04461-5

**Published:** 2025-11-07

**Authors:** Taofiq Adedeji Adeyemo, Sarah Greenley, Fiona Ware, Farzana Haque, Anthony Maraveyas, William Stephen Jones

**Affiliations:** 1 Centre of Excellence for Data Science, Artificial Intelligence and Modelling (DAIM), Faculty of Science and Engineering, University of Hull, Hull, UK; 2 Hull York Medical School, University of Hull, Cottingham Road, Hull, HU6 7RX UK; 3 Academic Library Services, University of Hull, Hull, UK; 4 Queens Centre for Oncology and Haematology, Castle Hill Hospital, Hull University Teaching Hospitals NHS Teaching Trust, Hull, UK

**Keywords:** Intracranial haemorrhages, Glioma, Anticoagulants, Predictive model, Prediction

## Abstract

**Background:**

People with glioma (PwG), a type of brain tumour, have an elevated risk of developing venous thromboembolism (VTE). When VTE occurs, anticoagulant therapy is typically initiated, and in some cases, it may be prescribed prophylactically. However, these patients are also at risk of intracranial haemorrhage (ICH) as a complication of anticoagulation. Despite the clinical importance of this risk-benefit balance, it remains unclear whether predictive tools exist to guide anticoagulation decisions in this population.

**Methods:**

We conducted a scoping review to determine whether predictive models exist for estimating the risk of intracranial haemorrhage (ICH) in people with glioma (PwG) receiving anticoagulant therapy. For any models identified, we assessed their methodological quality and predictive performance. Our search included MEDLINE, EMBASE (via Ovid), and the Cochrane Library, covering publications up to 29 November 2024. Studies were eligible if they employed predictive modelling to assess ICH risk in anticoagulated PwG. Two reviewers independently screened studies and extracted data. We used the PROBAST tool to evaluate model quality.

**Results:**

Of the 1,585 articles screened, none met the inclusion criteria. Although some studies reported on ICH risk in PwG, none developed or validated predictive models tailored to this clinical context. One excluded study provides conceptual insights that may inform future modelling efforts.

**Conclusions:**

The absence of these models underscores a critical gap in neuro-oncology research and highlights the urgent need for targeted model development to support anticoagulation decision-making in PwG.

**Supplementary Information:**

The online version contains supplementary material available at 10.1186/s12883-025-04461-5.

## Introduction

Primary malignant brain tumours have an annual incidence of approximately 7 per 100,000, People with Glioma (PwG) accounting for 80–85% of these tumours in adults [1, 2]. These people face both thrombotic and haemorrhagic complications, with venous thromboembolism (VTE) affecting up to 30% of patients and significantly impacting survival outcomes [2–7]. Glioma is recognised as one of the most prothrombotic cancers, with mechanisms including tumour-related activation of the coagulation cascade, release of procoagulant microvesicles, and abnormal tumour vasculature contributing to this risk [2, 3, 7–9]. Several studies [6, 10] report that the cumulative incidence of VTE in PwG approaches 24% within the first year post-diagnosis, with most events occurring in the first three months after surgery.

Although anticoagulation therapy can improve outcomes in PwG with VTE [4], it also elevates the risk of intracranial haemorrhage (ICH), a complication strongly associated with poor survival [2, 5, 11–13]. In one retrospective analysis, PwG who developed VTE had a median overall survival of 14 months, compared to 19 months in those without VTE, further underscoring the clinical importance of thromboprophylaxis [14]. This risk is particularly pronounced in high-grade gliomas, where aggressive tumour biology and abnormal vascular features predispose patients to spontaneous or treatment-related ICH [15, 16]. Multiple Meta-analyses and cohort studies consistently report a significantly higher ICH risk among anticoagulated PwG compared to those not receiving anticoagulation [2, 5, 13].

Making clinical decision about anticoagulation in PwG is challenging, as it requires careful consideration of multiple factors, including tumour characteristics, timing of surgical interventions, platelet levels, functional status, and the balance between thrombotic and the risk of haemorrhage [17]. Also, the brain tumour microenvironment itself adds to this complexity, with abnormal vasculature, increased vessel density, and glioma infiltration of surrounding tissues predisposing patients to bleeding even in the absence of anticoagulation [2, 4, 5].

The absence of validated, glioma-specific tools for predicting ICH leaves clinicians reliant on retrospective studies, expert consensus, and individual judgement [18–20]. This complexity is further compounded by the diverse biological behaviour of glioma and individual variation in how PwG metabolise anticoagulants [19, 21, 22]. Moreover, choosing between low molecular weight heparin and direct oral anticoagulants (DOACs) introduces additional challenges, as emerging evidence suggests DOACs may carry a lower risk of ICH, although the data remain conflicting [23]. This issue is of more relevance as the population ages since anticoagulation is frequently indicated in PwG for VTE prophylaxis and common comorbidities such as atrial fibrillation, further underscoring the need for reliable ICH risk prediction [5, 13, 24].

Predictive models offer a promising approach for navigating this complexity of anticoagulation decisions in PwG. These tools have demonstrated good results in areas related to tumour grading, genetic profiling, and prognosis [25–28]. Their ability to integrate diverse clinical and image inputs and to capture non-linear relationships makes them potentially well-suited for risk prediction. With appropriate training and validation, they could support individualised anticoagulation decisions in this high-risk population [29].

In this scoping review we aim to: (i) determine whether any predictive models currently exist for predicting ICH in PwG receiving anticoagulation therapy; (ii) appraise the methodological quality and clinical relevance of any identified models; and (iii) map critical gaps in the current evidence base to guide future model development. By conducting a structured and comprehensive literature review, we aim to highlight key research gaps and contribute to the development of predictive tools that can guide personalised anticoagulation decisions in patients with glioma.

## Methods

### Study design

The review was conducted following the Preferred Reporting Items for Systematic Reviews and Meta-Analyses extension for Scoping Reviews (PRISMA-ScR) guidelines [30] to ensure methodological transparency.

### Eligibility criteria

Studies were eligible for inclusion if they met the following criteria: the population consisted of PwG, there was an anticoagulation therapy intervention, the incidence and risk of ICH were reported as an outcome, and a predictive model was implemented. Additionally, the studies had to be full-text, peer-reviewed articles published in English. Studies that did not meet these inclusion criteria were excluded; no further exclusion criteria were deemed necessary.

### Search strategy

We conducted a structured literature search using MEDLINE and EMBASE via the Ovid platform, as well as the Cochrane Library, which include the Cochrane Database of Systematic Reviews and CENTRAL. The search covered publications up to 29 November 2024. The strategy was developed by TA, WSJ, FH & AM iteratively based on relevant terms identified in key papers, including existing reviews. These terms were expanded using controlled vocabularies such as MeSH (for MEDLINE) and Emtree (for EMBASE), alongside relevant free-text keywords. The final strategy focused on three main concepts: glioma as the population, anticoagulation as the intervention, and ICH as the outcome. Two experienced information specialists FW, and SG, reviewed the final search terms for completeness and accuracy. The complete search strategy is provided in Supplementary Appendix 1. As a limitation, the search was restricted to English-language publications and did not include supplementary citation chaining, grey literature, or trial registries, which may have led to the omission of potentially relevant studies.

### Study selection

Search results were imported into Covidence, where duplicate records were removed. Titles and abstracts were screened independently by two reviewers [TA & WSJ], and the full texts of potentially eligible articles were planned to be assessed in the same way. Any disagreements were discussed and resolved by consensus, with input from a third reviewer [FH] where needed.

Citation chaining and grey literature searches were planned to be conducted for included studies to identify additional relevant publications. The review focused on peer-reviewed publications to maintain consistency and ensure quality across the dataset.

### Data extraction

Data extraction was planned to be conducted by two independent reviewers using a structured approach. The key fields included study design, publication year, population characteristics, model type, variables used, outcome measures, and reported performance metrics. In cases of disagreement, a third reviewer would have mediated the discussion to reach consensus.

### Quality assessment

#### Risk of bias

Our protocol specified that we would use the PROBAST tool for evaluating prediction models. This tool remains relevant as frameworks for assessing the methodological quality of future studies in this area [31, 32].

## Results

**Fig. 1:**
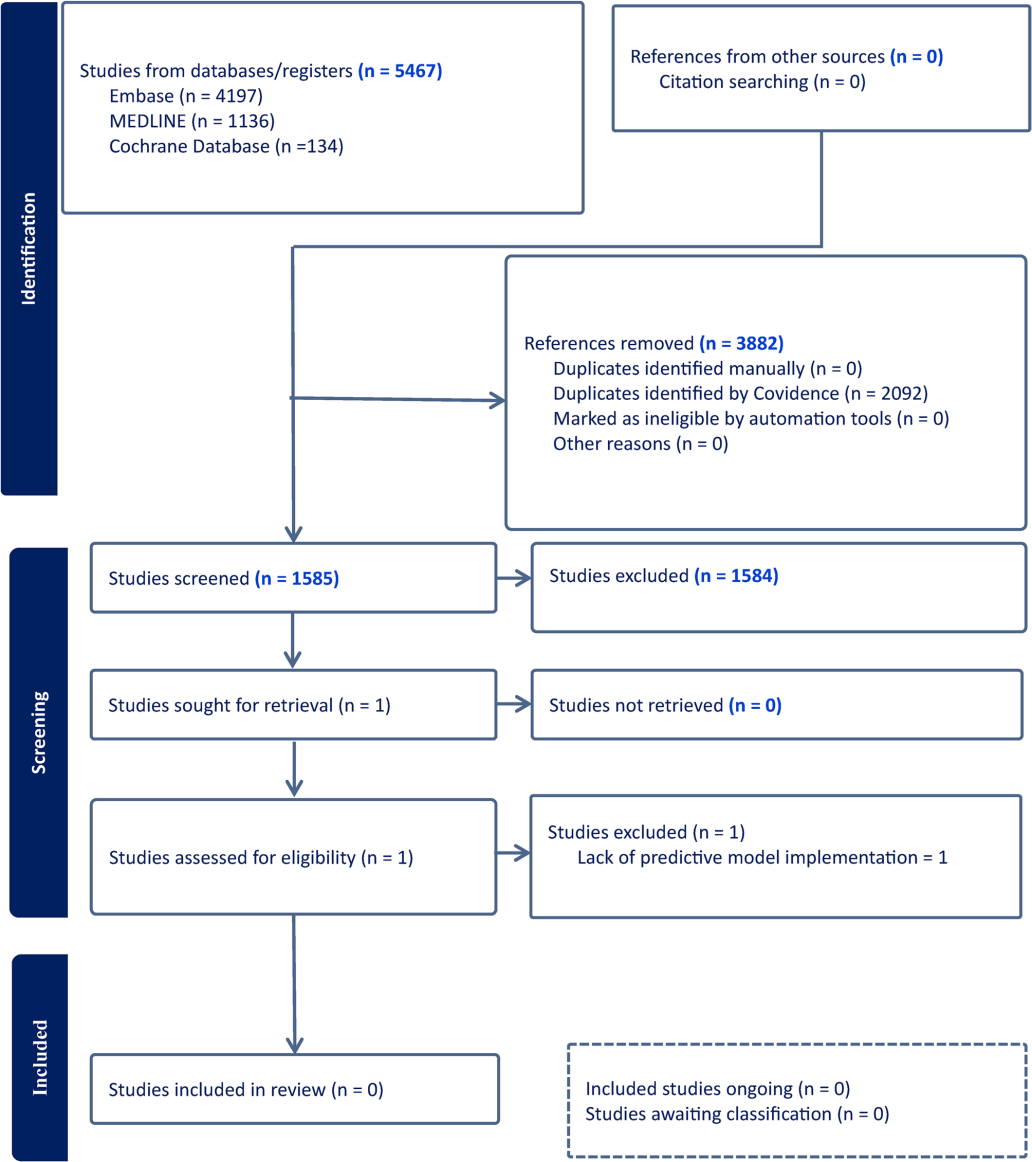
PRISMA flow diagram and results from literature screening

A total of 5,467 records were identified from EMBASE, MEDLINE, and the Cochrane Database. After removing duplicates, 1,585 unique articles were screened. Following title, abstract, and full-text review, no studies met the eligibility criteria. The full screening process is summarised in the PRISMA flow diagram (Fig. 1).

## Discussion

In this study, we aimed to determine whether any statistical or machine learning (ML) models exist for predicting the risk of intracranial haemorrhage (ICH) in people with glioma (PwG) receiving anticoagulation therapy. We also sought to evaluate the methodological quality and clinical relevance of any identified models and to map key evidence gaps to inform future model development. We screened 1,585 records from MEDLINE, EMBASE, and the Cochrane Library; however, no studies met our inclusion criteria. This absence highlights a fundamental gap in the literature. Given the aging population and increasing use of anticoagulation for both VTE and comorbidities such as atrial fibrillation [1], the development of a reliable, glioma-specific ICH prediction tool is very important.

Although no eligible predictive models were identified, one excluded study applied the PANWARDS score which was originally developed for atrial fibrillation to a glioma cohort, offering only limited exploratory insight [1]. In a retrospective analysis of 133 patients with high-grade glioma receiving enoxaparin, a higher rate of major ICH was observed compared to non-anticoagulated patients (14.7% vs. 2.5%; *P* =.036). All ICH events occurred in patients with PANWARDS scores ≥ 25 (sensitivity 100%, specificity 40%), yet the study did not involve model development or validation specific to PwG and therefore did not meet our inclusion criteria. While variables included in PANWARDS (platelet count, albumin, comorbidities) [33] may inform future work, any tool for PwG must go further and incorporate glioma-specific factors such as tumour grade, size, and location. Moreover, the low specificity of PANWARDS also limits its clinical utility, highlighting the need for models tailored to the unique biology and treatment context of PWG receiving anticoagulation.

To address this gap, future research should aim to integrate diverse data types which include glioma-specific factors, clinical, radiological, radiomic, and genetic features to inform robust predictive models. Advanced modelling techniques such as deep learning are particularly well suited to maximising the value of multidimensional datasets because they can learn complex, non-linear associations across different data modalities [34–36]. Convolutional neural networks (CNNs), which form the foundation of many deep learning approaches, are especially effective for medical imaging [37]. They can automatically detect and combine spatial patterns such as tumour boundaries, oedema, and vascular abnormalities, which are often difficult to capture with traditional statistical models. Beyond simple feature detection, CNN-based architectures such as U-Net, a widely used medical image segmentation network, extend this capability to image segmentation, enabling precise delineation of tumour and peri-tumoural regions [38]. These segmented regions can then be quantified through radiomics to extract detailed descriptors of texture, intensity, and shape, which, when integrated with clinical data, provide a rich representation of the factors that may influence ICH risk [39].

The development of such models must be guided by stakeholder engagement to ensure clinical relevance and practicality. Clinicians, patients, IT teams, and policymakers should be involved in defining the target product profile (TPP) for these models, ensuring they address real-world needs and constraints [40, 41]. While the exact model architecture will depend on the characteristics of future datasets, candidate approaches may include Random Forest, Decision Trees, Support Vector Machines, and neural networks. These methods are capable of handling high-dimensional, non-linear data and can be evaluated comparatively in future studies. Importantly, explainable AI techniques should be prioritised to enhance clinical interpretability, such as highlighting feature importance and decision pathways. We also recommend using established frameworks like PROBAST to ensure methodological rigour and support reproducibility in predictive modelling efforts.

While this study identified no existing models for predicting ICH risk in PwG on anticoagulation, it highlights a critical unmet need and provides a roadmap for future research.

The absence of existing predictive models may reflect several barriers. Glioma is a rare tumour, and the subset of patients receiving anticoagulation is even smaller, which limits the availability of large, representative datasets [42, 43]. Furthermore, it displays pronounced genetic and phenotypic heterogeneity, presenting significant challenges for establishing consistent predictors across cohorts [44]. Additionally, modelling ICH risk requires approaches capable of accounting for complex variable interactions and non-linear relationships, which traditional statistical methods may not fully capture [45]. These challenges highlight the need for advanced modelling techniques and harmonised data collection in future research.

In the absence of reliable prediction tools, clinicians must make anticoagulation decisions in PwG based on limited or generalised evidence. This creates significant uncertainty, particularly when trying to balance the known thrombotic risk against the potentially fatal consequences of ICH. The absence of validated models leaves a critical gap in supporting safe, individualised treatment planning. Addressing this gap through targeted predictive model development should therefore be treated as a research priority with direct clinical relevance.

To move the field forward, we propose a conceptual framework for developing predictive models using data from glioma with and without ICH. This would involve collecting high-quality clinical, radiological, and genomic data, followed by preprocessing and segmentation of imaging data to extract radiomic features. These variables would then be used to train a predictive model capable of estimating individualised ICH risk. This structured approach offers a practical foundation for future model development tailored to this high-risk population.

While the potential development of these models is important, they are unlikely to influence clinical decisions once VTE has been confirmed, as anticoagulation is typically initiated [46]. In these cases, the immediate need to manage thrombotic risk takes precedence. The primary value of our proposed model lies in prophylactic contexts, where decisions regarding anticoagulation are more discretionary [47]. These include determining the timing of anticoagulation around neurosurgical procedures, selecting appropriate agents and dosing strategies, and establishing the intensity of monitoring required [47]. Models designed for these scenarios could support more personalised and safer prevention strategies, aligning with current clinical practice and addressing a recognised gap in evidence-based decision-making.

The review used three major databases: MEDLINE, EMBASE and the Cochrane Library, which are widely regarded as principal sources of biomedical literature. While this approach ensured robust coverage of high-quality studies, it may have excluded relevant work indexed in other databases or published in languages other than English. Grey literature was not considered, consistent with our focus on peer-reviewed publications most relevant for clinical practice. Citation searching was not applicable, as no eligible studies were identified to provide a reference base. These boundaries were applied to maintain methodological consistency and transparency. Taken together, the absence of eligible studies indicates that predictive models for intracranial haemorrhage in glioma remain underdeveloped rather than reflecting a shortcomings in the search strategy.

## Conclusion

This review identifies a critical gap in the literature: the absence of predictive models for ICH risk in PwG receiving anticoagulation therapy. Consequently, clinicians must rely on limited or nonspecific guidance, which may result in inconsistent or suboptimal care for this vulnerable population. This situation underscores the urgent need for tools to support personalised decision-making in this high-risk group.

Future research must prioritise a multimodal approach, combining clinical, genetic, and radiological data to reflect the complex interplay of factors influencing ICH risk in glioma. Advanced modelling techniques, including machine learning, will be crucial to detect patterns within these high-dimensional datasets that conventional methods may overlook. Although tools like the PANWARDS score have identified potentially relevant variables, their lack of validation or specificity for glioma means that bespoke models are urgently needed.

However, technical innovation alone is not enough. Clinicians, patients, and regulators must collaborate to ensure these models are practical, clinically relevant, and aligned with real-world needs. Tools like TPPs can help define essential features, balancing predictive accuracy with usability and cost [41].

Addressing these challenges will enable the development of models that support clinicians and patients in making informed, personalised decisions, thereby reducing preventable harm and improving outcomes. Although the path forward is complex, the potential to transform care for PwG is substantial, offering the opportunity to resolve current gaps in evidence-based practice.

## Supplementary Information

Supplementary Material 1.

Supplementary Material 2.

## Data Availability

No datasets were extracted, as no studies met the inclusion criteria after full-text screening. The search strategy and screening process are available in the supplementary material.
