# Supplementary material for: Evidence gap in predicting intracranial haemorrhage risk in people with glioma on anticoagulants: a scoping review
Source: BMC Neurol. 2025 Nov 7;25:458. doi: 10.1186/s12883-025-04461-5 (PMC12595886; doi:10.1186/s12883-025-04461-5)
Supplement: Supplementary file 2 [file 12883_2025_4461_MOESM2_ESM.docx]

# Supplementary Material

## Appendix 1: Search Strategy

Scoping Review Search Strategy

# Proposed Research Questions:

# What is the predictive performance of predictive models in assessing intracranial bleeding risk among people with glioma receiving anticoagulant therapy?

# Review Study Design

# This study was conducted as a scoping review, initially registered on PROSPERO (CRD42024615118) and reported in accordance with the PRISMA guidelines.

# Reviewers

The study involved collaboration among me (Taofiq Adeyemo), Dr. Will Jones, and Dr. Farzana Haque. Dr. Jones and I independently screened the articles, with Dr. Haque serving as an independent arbitrator to resolve discrepancies in screening decisions.

Author Contributions

- Taofiq Adeyemo conceptualised the study, collected data, screened articles, and drafted the manuscript.

- Dr. Will Jones screened articles, provided methodological guidance, and reviewed the manuscript.

- Dr. Farzana Haque acted as a third-party mediator for resolving conflicts in article selection and reviewed the final manuscript.

# Databases: 

The following databases were searched:

MEDLINE, EMBASE, and COCHRANE Database.

# Keywords and Search Terms 

## MEDLINE and EMBASE:

- exp Brain Neoplasms

- exp Glioma/

- (brain metasta* or glioma* or meningioma* or glioblastom* or oligodendrogliom* or astrocytoma* or oligoastrocytoma* or Ependymoma* or Brain cancer* or brain tumo*).ti,ab,kw,kf.

- or/1-3

- exp Anticoagulants/

- (anticoagula* or anti-coag* or anti coag* or warfarin or heparin or low molecular weight heparin or lmwh or enoxaparin or oral anticoag* or blood thin* or DOAC* or direct oral anticoagulan* or vitamin K antagonist or thrombin inhibit*).ti,ab,kw,kf.

- 5 or 6

- exp Intracranial Hemorrhages/

- exp Cerebral Hemorrhage/

- (intracranial hemorrhag* or intracranial haemorrhag* or intracranial bleed* or cerebral hemorrhag* or cerebral haemorrhag* or cerebral bleed*).ti,ab,kw,kf.

- or/8-10

- 4 and 7 and 11

## COCHRANE DATABASE

1	MeSH descriptor: [Brain Neoplasms] explode all trees

2	MeSH descriptor: [Glioma] explode all trees

3	(brain metasta* or glioma* or meningioma* or glioblastom* or oligodendrogliom* or astrocytoma* or oligoastrocytoma* or Ependymoma* or Brain cancer* or brain tumo*):ti,ab,kw (Word variations have been searched)

4	MeSH descriptor: [Anticoagulants] explode all trees

5	((anticoagula* or anti-coag* or anti coag* or warfarin or heparin or low molecular weight heparin or lmwh or enoxaparin or oral anticoag* or blood thin* or DOAC* or direct oral anticoagulan* or vitamin K antagonist or thrombin inhibit*)):ti,ab,kw (Word variations have been searched)

6	MeSH descriptor: [Intracranial Hemorrhages] explode all trees

7	MeSH descriptor: [Cerebral Hemorrhage] explode all trees

8	((intracranial hemorrhag* or intracranial haemorrhag* or intracranial bleed* or cerebral hemorrhag* or cerebral haemorrhag* or cerebral bleed*)):ti,ab,kw (Word variations have been searched)

9	#1 or #2 or #3

10	#4 or #5

11	#6 or #7 or #8

12	#9 and #10 and #11

 

The focus was on articles written in English, covering the period from the inception of MEDLINE and EMBASE until 29 November 2024.

## Tools:

Covidence was used to streamline the production of this review. This web-based software facilitated collaboration among review team members, allowing for efficient screening of citations, full-text reviews, risk of bias assessments, and data extraction. Covidence also automatically populated a PRISMA flow diagram to document the review process (Ahmad, 2023).

## Inclusion Criteria: 

The inclusion criteria for screening and selecting studies for this review will be as follows:

- Population: Studies on people with glioma receiving anticoagulant therapy.

- Outcome: Studies assessing intracranial haemorrhage risk prediction using predictive models.

- Study Design: Studies employing predictive models.

- Language: English language studies only.

- Publication Date: Inception to 29 November 2024.

## Exclusion Criteria: 

- Studies not conducted on human subjects.

- Studies not published in English.

- Studies focusing on non-brain cancer patients.

- Studies not assessing intracranial bleeding risk prediction in PwG.

- Studies not employing predicitve models for risk assessment in PwG on anticoagulants.

- Studies with a substantial risk of bias or methodological flaws.

- Duplicate publications or multiple reports of the same study.

## Risk Of BIAS

## The risk of bias assessment was initially planned to use the Prediction model Risk Of Bias Assessment Tool (PROBAST). This tool is specifically designed to assess the quality of primary studies that develop, validate, or update multivariable models (Karel G.M. Moons, 2019). However, as no studies met the inclusion criteria for this review, the PROBAST tool was eventually not used.

## References

Ahmad, Bilal. “Streamlining Research: Unveiling the Power of Evidence Synthesis with Covidence.” Covidence, 18 July 2023, www.covidence.org/blog/streamlining-research-unveiling-the-power-of-evidence-synthesis-with-covidence/.

Brown.edu. “LibGuides: Guide to Searching: Citation Searching.” Brown.edu, 2013, libguides.brown.edu/searching/citation.

Haddaway, Neal R., et al. “Citationchaser: A Tool for Transparent and Efficient Forward and Backward Citation Chasing in Systematic Searching.” Research Synthesis Methods, vol. 13, no. 4, 7 May 2022, pp. 533–545, https://doi.org/10.1002/jrsm.1563.

Moons, Karel G.M., et al. “PROBAST: A Tool to Assess Risk of Bias and Applicability of Prediction Model Studies: Explanation and Elaboration.” Annals of Internal Medicine, vol. 170, no. 1, 1 Jan. 2019, p. W1, https://doi.org/10.7326/m18-1377.
